# Methods Used in the Development of Common Data Models for Health Data: Scoping Review

**DOI:** 10.2196/45116

**Published:** 2023-08-03

**Authors:** Najia Ahmadi, Michele Zoch, Patricia Kelbert, Richard Noll, Jannik Schaaf, Markus Wolfien, Martin Sedlmayr

**Affiliations:** 1 Institute for Medical Informatics and Biometry Carl Gustav Carus Faculty of Medicine Technische Universität Dresden Dresden Germany; 2 Fraunhofer Institute for Experimental Software Engineering IESE Kaiserslautern Germany; 3 Institute of Medical Informatics Goethe University Frankfurt University Hospital Frankfurt Germany; 4 Center for Scalable Data Analytics and Artificial Intelligence Dresden/Leipzig Germany

**Keywords:** common data model, common data elements, health data, electronic health record, Observational Medical Outcomes Partnership, stakeholder involvement, Data harmonisation, Interoperability, Standardized Data Repositories, Suggestive Development Process, Healthcare, Medical Informatics

## Abstract

**Background:**

Common data models (CDMs) are essential tools for data harmonization, which can lead to significant improvements in the health domain. CDMs unite data from disparate sources and ease collaborations across institutions, resulting in the generation of large standardized data repositories across different entities. An overview of existing CDMs and methods used to develop these data sets may assist in the development process of future models for the health domain, such as for decision support systems.

**Objective:**

This scoping review investigates methods used in the development of CDMs for health data. We aim to provide a broad overview of approaches and guidelines that are used in the development of CDMs (ie, common data elements or common data sets) for different health domains on an international level.

**Methods:**

This scoping review followed the PRISMA-ScR (Preferred Reporting Items for Systematic Reviews and Meta-Analyses extension for Scoping Reviews) checklist. We conducted the literature search in prominent databases, namely, PubMed, Web of Science, Science Direct, and Scopus, starting from January 2000 until March 2022. We identified and screened 1309 articles. The included articles were evaluated based on the type of adopted method, which was used in the conception, users’ needs collection, implementation, and evaluation phases of CDMs, and whether stakeholders (such as medical experts, patients’ representatives, and IT staff) were involved during the process. Moreover, the models were grouped into iterative or linear types based on the imperativeness of the stages during development.

**Results:**

We finally identified 59 articles that fit our eligibility criteria. Of these articles, 45 specifically focused on common medical conditions, 10 focused on rare medical conditions, and the remaining 4 focused on both conditions. The development process usually involved stakeholders but in different ways (eg, working group meetings, Delphi approaches, interviews, and questionnaires). Twenty-two models followed an iterative process.

**Conclusions:**

The included articles showed the diversity of methods used to develop a CDM in different domains of health. We highlight the need for more specialized CDM development methods in the health domain and propose a suggestive development process that might ease the development of CDMs in the health domain in the future.

## Introduction

### Rationale

Integration of heterogeneous data is a ubiquitous topic in modern medicine. The arising large variety of data has the potential to provide in-depth insights about different aspects of clinical care and can lead to improvements in health care [1,2]. Yet, challenges, such as the identification and access of relevant data, the association between different data sources, and the assurance of data quality given the structural variations among data sources, still pose major barriers [3,4]. Common data models (CDMs) provide the possibility of harmonizing data from disparate sources, storing information in a standard structure by defining the syntax and semantics of data, and enabling operations on data using standard analysis methods [5]. In particular, a CDM contains a unified set of metadata, allowing data and its information content to be shared across applications and institutional borders, and thus enabling harmonized data integration and analysis on an international scale [6].

In the health domain, there are different types of CDMs (eg, CDMs for harmonization and storage of electronic health record–based patient data). An example is the Observational Medical Outcomes Partnership Common Data Model (OMOP CDM) developed by the Observational Health Data Science and Informatics (OHDSI) community, which ensures homogeneous storage of observational health care data across different databases with similar formats and terminologies [7]. There are also further CDMs for clinical data, like Sentinel CDM, Clinical Data Interchange Standards Consortium (CDISC) Study Data Tabulation Model (SDTM), and National Patient-Centered Clinical Research Network (PCORnet) [8], and data warehouse models, like Informatics for Integrating Biology and the Bedside (i2b2) [9]. Moreover, some CDMs define the data from patient cohorts and describe a medical specialty or a group of diseases. For example, there are specific CDMs for the domain of rare diseases [10,11] or radiology [12]. Overall, there is a large variety of CDMs in the literature for common, rare, and context-specific medical examinations, and each of them follows a more self-defined development process.

As described by Melles et al [13], a practical design meets the users’ needs. While designing a CDM in the health domain, in addition to the developers (ie, IT staff and computer scientists), the primary stakeholders (ie, patients and clinicians) are particularly interested in the outcome. It is therefore recommended to include them in the design process as early as possible [13,14]. In addition to the stakeholders, the medical context is also quite complex and requires extensive medical and technical expertise to ensure the usefulness of the model after its development. This is why the development process of a CDM is critical and a comprehensive development method or guideline is necessary.

Studies, such as those by Gericke and Blessing [15] and Bobbe et al [16], have already tried to determine the commonalities and differences in development processes across disciplines. Bobbe et al [16] performed a comparison of design models from academic theory and professional practice, and discussed 8 types of design processes. In particular, the basic design cycle, V design process, human-centered design, hypercyclic design, Munich procedural model, double diamond model, frog model, and IDEO model were presented. Additionally, Melles et al [13] introduced categories for models, namely, whether a model is activity-based or stage-based, solution-oriented or problem-oriented, and design-focused or project-focused.

However, given the complexity of the health domain and the importance of many stakeholders taking part in the process, it might be difficult to transfer models from other disciplines. This is why we aim to derive such a process and review the available CDM instances in the domain. Exemplarily, the results of this scoping review will be integrated into the design and development of a CDM for the SATURN (“Smartes Arztportal für Betroffene mit unklarer Erkrankung” [“Smart physicians’ platform for patients with unclear diseases”]) Project in the future [17]. This project aims to develop an artificial intelligence–based diagnosis support tool for primary care physicians. With the help of user-centered design, the requirements of a decision support tool, especially for noncharacteristic symptoms, will be studied. The medical focus is on the diagnosis of unclear and rare medical conditions. This is why, in this review, we focus on the similarities between the CDM development methods in rare medical conditions and common medical conditions in order to determine whether the methods for common medical conditions can be adopted for rare medical conditions as well. On a technical level, rule-based systems, machine learning, and case-based reasoning will be implemented. As part of this project, CDMs for 3 groups of rare diseases, namely, endocrinology, gastroenterology, and pneumology, will be developed.

Our review contributes to the analysis of CDM development methods in the health domain on an international scale and aims to explore the actual involvement of stakeholders, especially medical experts, in the development process. To the best of our knowledge, this is the first scoping review focusing on CDM development methods in the health domain.

### Objectives and Research Questions

This scoping review has been conducted to provide an overview of the methods used for the initial and further development of CDMs in the health domain. We divided the overall development process into conception, users’ needs collection (eg, collection of evidence, review of the literature, and guidelines), and implementation, as well as individual evaluations within the phases. We consider the conception phase as an initial step, where the CDM is theoretically designed along with stakeholders. Subsequently, the essential elements previously identified are gathered in the “users’ needs collection” phase. The finalized process, in which the conceptualized model is implemented and ready-to-use, is termed the implementation phase.

According to the rationale and objective explained above, this scoping review examines the following questions:

1. How are CDMs methodically developed in the health domain? What requirement analysis methods, design processes, and validation methods were used?
2. How or when do stakeholders, especially medical experts, get involved in the development process?
3. How can the CDM development methods be classified based on their requirement analysis methods, design processes, validation methods, and model type?

## Methods

### Protocol and Registration

To ensure methodological quality, this scoping review has followed the Preferred Reporting Items for Systematic Reviews and Meta-Analyses extension for Scoping Reviews (PRISMA-ScR) checklist [18]. According to this checklist, we published and registered the review protocol [19]. Out of the 22 items of the PRISMA checklist, 20 have been considered in this review (Multimedia Appendix 1).

### Search Strategy

To achieve a comprehensive query, an initial search was performed in PubMed with the term “common data model.” Six randomly chosen articles matching the topic were analyzed [10-12,20-22]. The keywords associated with the articles listed in Table 1 were considered and subsequently tested in the query. The combination of terms that delivered the highest number of matching articles was included in our final search string.

Some studies used the term *data set* [11], and others defined alternative data elements that can be part of a data set or data model [10]; thus, to avoid the exclusion of certain studies, we jointly used the following terms in our search string: *common data model*, *common data element*, and *common data sets*. We also added the short forms of these terms in our search string and analyzed the relevance of the results by simply looking into the resulting literature. Additionally, we added the following terms in our search string to ensure that the included CDMs were developed within the health domain: medical, medicine, health, healthcare, health care, electronic health, clinical, and disease. The search string used in PubMed is presented in Table 2. It was developed as a combination of the mentioned terms, their possible variations, and where applicable, Medical Subject Headings (MeSH) [23]. The search strings used in the other 3 databases have been provided in Multimedia Appendix 2.

The query was designed and tested by the author NA and was approved by all coauthors. The resulting articles were added to Rayyan (Rayyan Systems Inc) [24] for further screening and annotation.

**Table 1:** Six randomly chosen articles for the construction of the search string and their keywords.

Article title	Keywords
The EPIRARE proposal of a set of indicators and common data elements for the European platform for rare disease registration [10]	Registries, common data elements, European platform, rare diseases, patient registration, and EPIRARE
A methodology for a minimum data set for rare diseases to support national centers of excellence for healthcare and research [11]	Common data elements, interoperability, metadata, minimum data set, national health program, and rare diseases
Development and validation of the Radiology Common Data Model (R-CDM) for the international standardization of medical imaging data [12]	*Metadata*, standardization,* *and radiology information system
Common data model for natural language processing based on two existing standard information models: CDA+GrAF [20]	Natural language processing, medical informatics, data model, information model, HL7 clinical document architecture, and ISO graph annotation format
Genomic common data model for biomedical data in clinical practice [21]	High-throughput nucleotide sequencing, data analysis, and observational study
Towards a newborn screening common data model: The Utah Newborn Screening Data Model [22]	Newborn screening, newborn screening laboratory information management system, common data model, interoperability, electronic data exchange, NBS, LIMS, and standards

**Table 2:** Search strings used to identify articles from PubMed.

Search aspects	Variations	Search string^a^
Common data model	Common data model (CDM), common data element (CDE), and common data sets (CDS)	(“common data model” AND CDM) OR (“common data element*” AND CDE) OR “Common Data Elements”[Mesh] OR “common dataset*” OR “common data set*”
Health care	Medical, medicine, health, healthcare, health care, electronic health, and disease	medical OR medicine OR “Medicine”[Mesh] OR health OR “Health”[Mesh] OR healthcare OR “health care” OR “electronic health” OR clinical OR disease OR “Disease”[Mesh]

^a^The common data model and health care search terms were combined with “AND.”

In particular, literature from 2000 to 2022 was considered, which is an extension of the previously published study protocol [19]. It is also noteworthy that the MeSH terms were only available in PubMed. The language of the articles was limited to English. Using the Boolean operators “AND” and “OR,” the systematic search was carried out in the following electronic databases: PubMed, Web of Science, Science Direct, and Scopus. The search was performed in March 2022. The publication date tag in PubMed and Web of Science was set to January 1, 2000, to March 15, 2022, and that in Science Direct and Scopus was set to 2000 to 2022 (it is not possible to specify the month and day in Science Direct and Scopus).

### Inclusion and Exclusion Criteria

**Figure 1:**
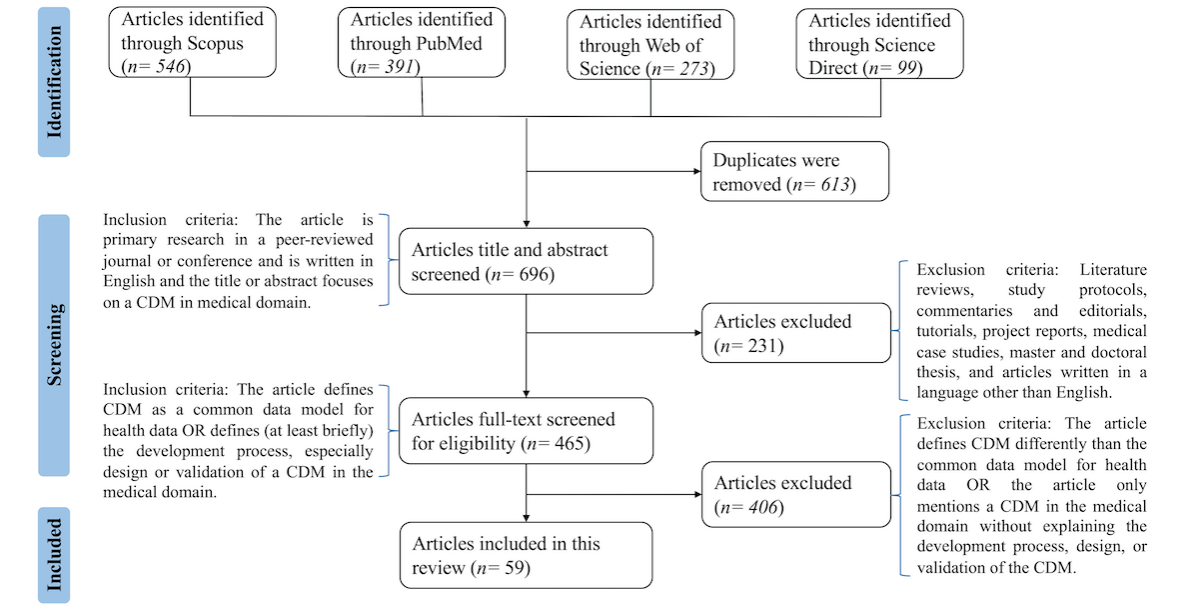
PRISMA (Preferred Reporting Items for Systematic Reviews and Meta-Analyses) flowchart showing the paper selection process and the inclusion and exclusion criteria. CDM: common data model.

The inclusion and exclusion criteria are summarized in Multimedia Appendix 3 and are visualized along with the number of outcome articles in Figure 1.

### Selection and Review of Articles

Duplicates were removed using the built-in function in Rayyan [24]. The process of deletion was monitored by the author NA. After eliminating duplicates, the selection of studies was performed in 2 steps. The title and abstract screening steps were performed by the authors in groups of two. The articles were tagged as “include,” “exclude,” or “maybe.” Tagged articles were decided upon based on the tags described in Table 3.

Disagreements were resolved by a third author. This process was initially carried out on 10% of the articles to confirm the accuracy of our inclusion and exclusion criteria, and clarify ambiguities. After the title and abstract screening, the full text of the included articles was screened by the authors, again in groups of two. The selected articles were included in the data extraction step.

**Table 3:** Description of tags used by the authors in the article screening process.

Author 1	Author 2	Decision
Include	Include	Included
Include	Exclude	Discuss and decide together
Include	Maybe	Include
Exclude	Maybe	Exclude
Maybe	Maybe	Discuss and decide together

### Data Charting and Extraction Process

A data charting table was developed and refined throughout the study, with several iterations. This table contained a list of items that were extracted from all included publications. All authors examined 10% of the articles for the defined data items and refined the data charting table, if necessary. The data charting table, including the extracted information from articles, is included in Multimedia Appendix 4.

For each article, we focused on 4 major aspects: (1) the meta information, such as DOI, authors, year, country, and project name, if applicable; (2) the medical condition for which the CDM was built, whether the condition is rare or common, the organ affected by the condition, and whether the condition is long term (longer than a year) or short term; (3) methodological information, such as requirement analysis, design, and validation process; whether the design process was linear or iterative; and advantages and disadvantages of the method, as stated in the respective article; and (4) information about stakeholder involvement. The extracted data elements, their categories, and their definitions are shown in Table 4.

**Table 4:** Data extraction sheet with specified elements, categories, and subcategories, including their definitions.

Category and subcategory		Definition
**Meta information**		
	DOI	A link to the article
	Author	First author’s name
	Publication year	Year of the publication date of the article
	Country of study	Country of the leading author’s affiliation
	Project name	If applicable; when the CDM^a^ study was part of a project/consortium
**Medical background**		
	Medical condition	Name of the medical condition for which the CDM was built
	Organ function	Organ affected by the medical condition
	Short-term/long-term condition	Short term: less than a year; long term: longer than a year
	Is the condition rare or common?	Is the medical condition considered rare or common based on its occurrence? Available answers: common medical condition, rare medical condition, and conditions that can be rare and common.
**Requirement analysis method**		
	Literature analysis	It includes searching in a variety of literature, such as extraction of frequent CDEs^b^ from real-world data, data harmonization across studies, multicenter longitudinal and observational studies, consensus documents and guidelines, primary outcome data of trials, review of instruments, and forms like report forms, users’ needs collection forms, etc.
	Interview/questionnaire	It includes expert interviews, focus group meetings, working group meetings, consensus meetings, workshops and discussions, and online surveys.
	Delphi	Delphi or modified Delphi was used. Delphi techniques involve experts evaluating complex issues iteratively, where knowledge is incomplete or uncertain. Typically, the response from the previous questionnaire is appended to the next questionnaire [25].
	Review of existing CDEs	When an existing CDE was validated/reviewed.
**Design**		
	Creation of new CDEs	If there were no CDEs in the domain and the experts tried to come up with some CDEs using literature in the field.
	Modification of existing CDEs	If existing CDEs in a disease domain were modified.
	Reuse of existing CDEs (without modification)	If existing CDEs in the domain were used without any modification.
**Validation**		
	External experts	It includes only external validation of any sort, such as public reviews on a website from experts or nonexperts in the field. Excluded are experts that were part of the conception process of the model.
	Others	Any other type of validation, such as internal reviews, working group consensus, etc.
**Model type**		
	Iterative	When at least one iterative process was performed during development of the CDM.
	Linear	When there was no iteration in the process.
**Stakeholder information**		
	Were stakeholders involved in the design process?	Yes/no
	Which stakeholders were involved?	Patients’ representatives, clinicians, domain experts, computer scientists, IT personnel, and registry staff
	When did they get involved in the process?	In users’ needs collection (when experts were involved in the preanalysis step, eg, collection of evidence, review of literature, guidelines, etc), in conception (when experts were involved in conception of the CDEs), in evaluation (when the model was evaluated via experts), and in implementation (when experts were involved in the implementation of the model).
	What was the nature of stakeholder involvement?	Through expert workshops, semistructured interviews, questionnaires, etc
**Pros and cons of methods as mentioned in the article**		
	Pros	Advantages of the method as stated in the article
	Cons	Disadvantages of the method as stated in the article

^a^CDM: common data model.

^b^CDE: common data element.

### Visualization and Summarization of Results

At the end of the data extraction, the data items collected in Table 4 were summarized and visualized. A flowchart according to the PRISMA-ScR guidelines was designed to show the article processing approach (Figure 1). Tables, timeline plots, histogram charts, pie plots, and scatter histograms were used to display the extracted data items. The graphics and the required analysis were performed using Python version 3.9.12 (Python Software Foundation), with matplotlib, pandas, and NumPy packages. The script used for the plots is publicly available [19,26].

First, we aimed for a broad overview of available CDMs and whether original CDMs were developed or existing CDMs were modified, as well as whether they focused on common or rare diseases and addressed a specific organ function. Second, to answer our first research question, we documented the medical domain of each article, whether the medical condition was considered as long term (more than a year) or short term (less than a year), and the affected organ as stated in the respective original article. To classify the development process of the CDMs, we documented 4 categories of data information for each article: requirement analysis, design, validation, and model type (Table 4). We categorized the methodology that was used for the requirement analysis (ie, why a CDM was needed), as well as the context to design a set of common data elements (CDEs). For validation, we distinguished between external evaluation and any other type of evaluation. The “other” category included the evaluations performed by the same clinical experts who were involved in the conception process, such as working group consensus, user evaluations, reviews performed via the members of the project, statistical tests, and pilot tests conducted within the project. Additionally, we investigated stakeholder involvement in the development stages in those studies and whether the studies followed an iterative or linear method of development. We used the advantages and disadvantages of the methods as stated in the articles (Table 4) and formulated them into a list of constraints in the area of CDM development to further highlight the need for streamlined methods. Finally, after analyzing the included CDMs, we summarized the most frequent methods used in the included literature in a suggestive development process that could be a reasonable basis to start with when developing a novel model.

## Results

### Selection of Articles

In total, we identified 1309 articles from PubMed, Web of Science, Science Direct, and Scopus search engines. From the identified articles, after duplicate removal, 695 articles were included in the title and abstract screening. Finally, 465 articles underwent full-text screening, and of these, 59 matched the full-text screening criteria of this review and were finally included. We excluded articles that did not describe the development or evaluation of a CDM in the health domain. Additionally, articles that were not publicly available and those in a language other than English were excluded. The article identification process along with the inclusion and exclusion criteria are shown in Figure 1.

**Figure 2:**
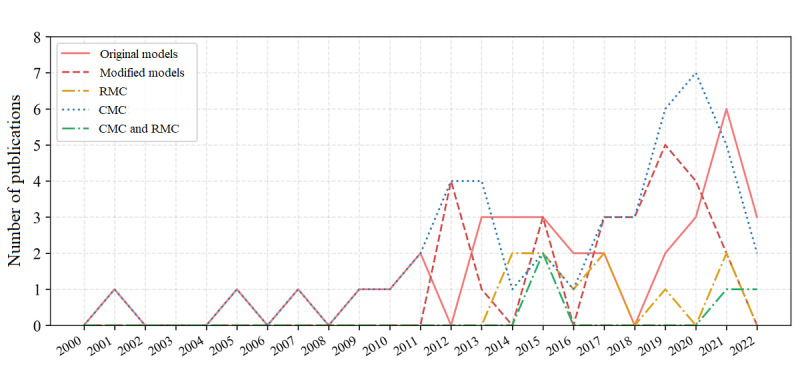
The number of publications focusing on common data model (CDM) development per year from 2000 to 2022. The line chart compares the number of articles developing original CDMs (original models) with the number of articles developing CDMs via modification of existing models (modified models), and compares the number of articles developing CDMs for rare medical conditions (RMCs), the number of articles developing CDMs for common medical condition (CMCs), and the number of articles developing CDMs for both kinds of conditions (CMCs and RMCs). In addition to the increase in the number of articles from 2011 in general, we can see that CDMs for rare diseases were only developed starting from 2014.

The selected articles defined CDMs, common data sets, or CDEs for common or rare medical conditions. All included articles were published between 2000 and 2022. As shown in Figure 2, the number of articles that focused on CDM development increased after 2011 and continued to increase in the last years.

### Country of Publication

We categorized the articles into countries based on the affiliation of the first author. Among the 59 articles, 26 (44%) were published in the United States, 8 (14%) were published in Canada, and 6 (10%) were published in Germany. The number of articles according to country is as follows: Belgium, 2 [27,28]; Canada, 8 [29-36]; China, 1 [37]; Denmark, 2 [38,39]; France, 2 [11,40]; Germany, 6 [41-46]; Italy, 1 [10]; Spain, 1 [47]; Republic of Korea, 1 [48]; Norway, 3 [49-51]; Switzerland, 1 [52]; Taiwan, 1 [53]; the Netherlands, 1 [54]; United Kingdom, 3 [55-57]; and United States, 26 [58-83].

### Medical Conditions and Their Domains

**Figure 3:**
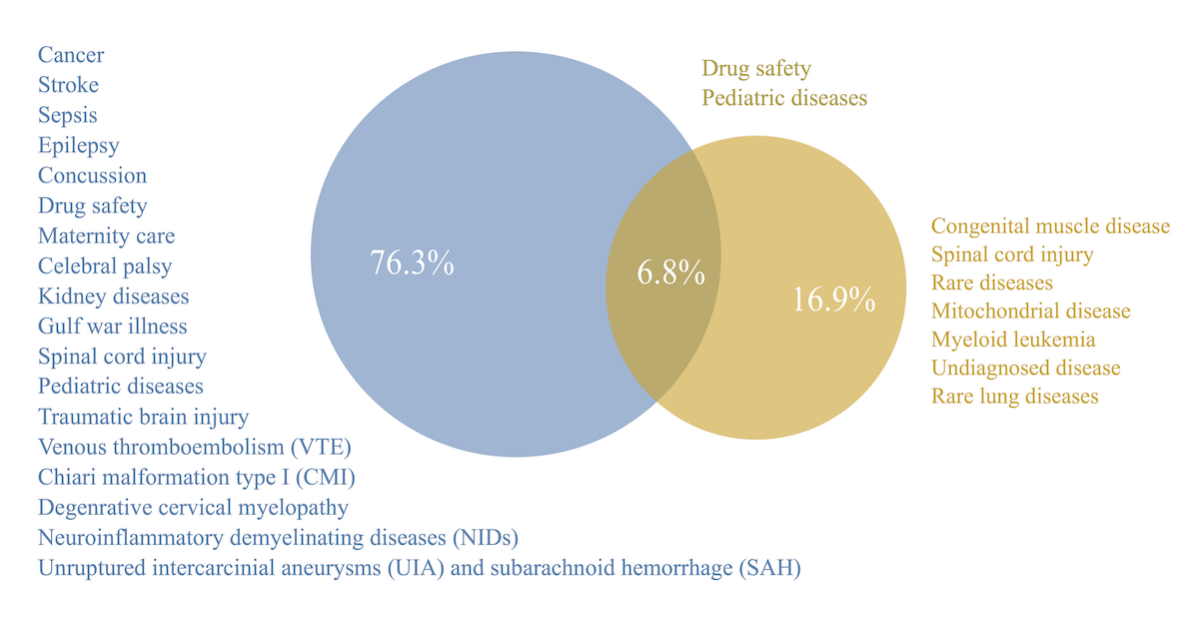
Characteristics of the included studies. A Venn diagram showing the proportions of identified common data models (CDMs) for common medical conditions (76.3%; blue), rare medical conditions (16.9%; golden yellow), and medical conditions that could fit into both categories (6.8%). Additionally, an aggregated list of medical conditions that CDMs were developed for in the studies is shown in 3 different colors according to their categories.

According to our research, CDMs were developed for a variety of medical domains in the past 22 years; however, we divided them into 3 categories, namely, rare, common, and rare and common (both). An aggregated list of the medical conditions and their domains is shown in Figure 3. A full list of the medical conditions extracted during this scoping review is shown in Multimedia Appendix 4. An organ function overview and the long- and short-term conditions are shown in Multimedia Appendix 5. Among these, 10 (17%) CDMs were designed for rare medical conditions, such as myeloid leukemia and rare lung diseases, and mitochondrial diseases [41,44-46,59]. Moreover, 1 CDM, namely, the CDM in the study by Berger et al [44], was designed for undiagnosed diseases in general.

Among the 59 articles, 45 involved the development of a CDM for common medical conditions. These included traumatic brain injury [27,28,30], spinal cord injury in children and youth [67], dental caries [68], sport-related concussion [65], cerebral palsy [29], degenerative cervical myelopathy [55], unruptured intracranial aneurysms and subarachnoid hemorrhage [32,42,55,60], Chiari malformation type I [63], breast implant [43], stroke [37], venous thromboembolism [33], pediatric epilepsy [61], pediatric critical illness [62], pregnancy drugs and treatments [49], sepsis [31], medication use in pregnancy and breastfeeding [40], degenerative cervical myelopathy [55], Gulf War illness [58], neuroinflammatory demyelinating disease [43], traumatic brain injury [27], and neurologic disorder and stroke [69]. Wandner et al [66] focused on clinical pain management, and Jaboyedoff et al [52] focused on pediatric diseases in general.

### Stakeholder Involvement

To investigate the involvement of stakeholders, we summarized at which particular stage they were involved in the CDM development process. Out of the 59 included articles, 54 (92%) mentioned at least one stakeholder in the design process. Additionally, we were interested in the different types of stakeholders that were involved, how they were involved, and at what stage of the process they typically got involved. As shown in Figure S1 in Multimedia Appendix 6, stakeholders were mostly involved in the initial stage, namely, the conception phase. Domain experts and clinicians were the most common stakeholders involved in the studies (Figure S2 in Multimedia Appendix 6). Additionally, while many different methods were used to involve the stakeholders, such as expert groups, surveys, consensus meetings, interviews, teleconferences, questionnaires, and workshops, “working group” was the most frequent method used (Figure S3 in Multimedia Appendix 6).

### Design Process

**Figure 4:**
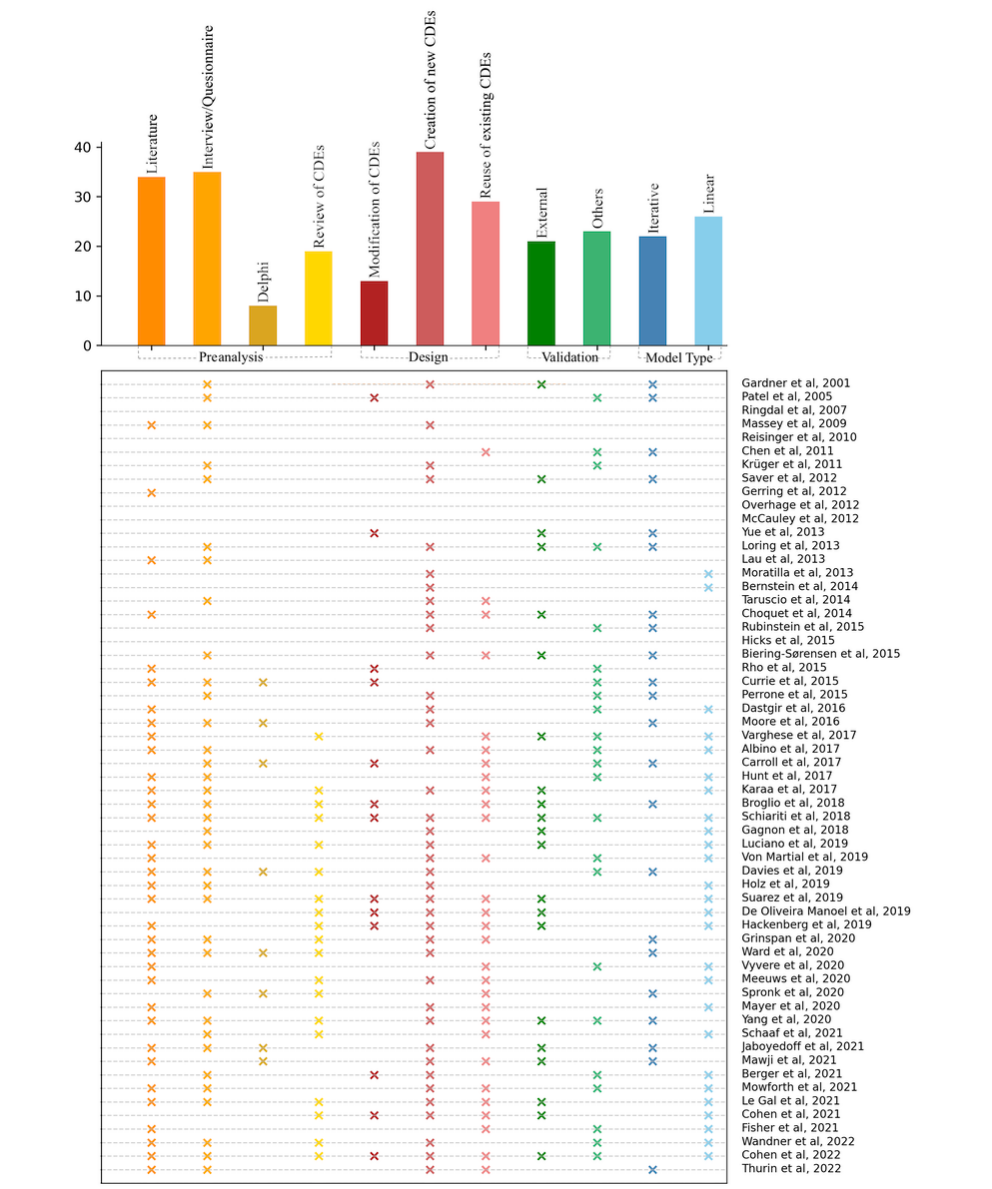
Methodological information on the articles [10,11,27-83]. The y-axis shows the list of articles by publication year. The x-axis shows the methodological categories. The scatter plot includes a cross mark when the Boolean is true for a specific article, for example, if the authors have used literature analysis as a preanalysis method, a cross (x) is added. The sum of cross marks in each column contributes to the bar size of the bar plot positioned on the x-axis. To improve visibility, each subcategory is shown with a different color. The subcategories of the same category are grouped via the same family of colors. CDE: common data element.

The methods used in the articles for designing a CDM were literature analysis, interview, Delphi, and review of existing CDEs. From our extraction table (Multimedia Appendix 4), we noted that 39 articles involved the definition of an original model/set of CDEs, 13 involved the modification of an existing set of CDEs, and 29 involved the use of an existing set of CDEs without any modifications. The external evaluation included web-based feedback, public review and comments, and feedback in a conference, among others. Finally, we found that 26 articles involved a rather linear design method and 22 others involved an iterative process. The list of articles that involved the use of each of these categories is shown in Figure 4. Detailed information is presented in Multimedia Appendix 4.

### Methodological Constraints Highlighted in Previous Studies

The included articles presented a range of constraints in the development process from the methods used in the different stages of the process to the applicability of the outcome elements. For example, Thurin et al [40] performed interviews with a single data access provider per data source and mentioned that other data access providers might conceptualize the data source differently. Additionally, they tested the applicability of the developed model only on the included data sources in the project. The model might require modification to use it with other data sources. The limited sample size used to test the developed model is a common problem in rare conditions [44] given the rarity of the disease. One of the limitations mentioned by Broglio et al [65] is that some of their developed CDEs require special expertise that might not be implementable in certain settings. Grinspan et al [61] mentioned that some subcategories of epilepsy syndrome were merged at a level higher into a single category, which might have led to reduced data resolution, although uphill mapping is often used, especially in the OMOP context [5]. Additionally, the elements considered do not cover every possible influencing element, and the source was limited to only US-based patients, which means the elements can differ once an international data level is considered. They also included CDEs that were documented as free text, and processing of such elements might require natural language processing applications. The authors also highlighted the possible bias caused by the methodology used for consensus and discussion, and the Delphi approach, focus groups, and interviews might have also influenced the outcome of the study.

### Essence of the CDM Development Process

**Figure 5:**
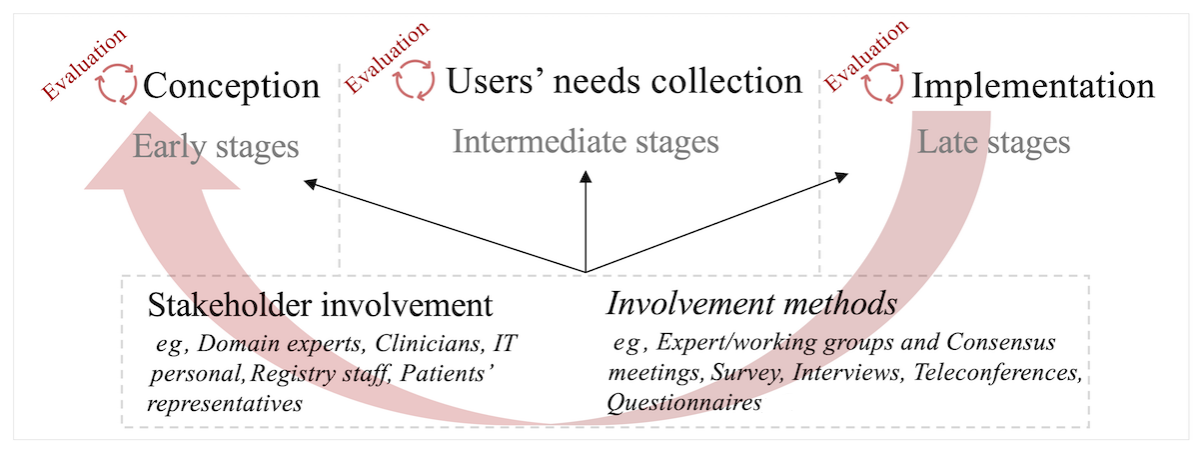
Summary of a basic common data model development process.

Our outcomes showed that a heterogeneous variety of methods or processes were used in CDM development in the included articles, which highlights the need for a more streamlined field-specific development method. Therefore, we summarized our analysis outcomes into a suggestive development process (Figure 5), considering the 3 development steps that have been identified from the included models in this study, namely, conception, users’ needs collection, and implementation. We suggest that evaluation and validation should be integrated into every stage of development, which gives the stages an iterative nature, and feedback should be integrated into the process as much as possible. We also emphasize the involvement of stakeholders in the process as early as possible and propose continuous involvement until the end of the development process because in every phase, questions might arise that need to be answered from different perspectives.

## Discussion

### Overview

One of the major challenges faced by CDM developers in the health domain is the lack of a comprehensive methodology or workflow to follow, which is also reflected in this review. The general models from industrial design and even academia (eg, the model introduced by Bobbe et al [16]) do not generally translate one-to-one to the health domain. The medical context is usually complex, and the involvement of stakeholders, such as clinicians, patients’ representatives, and IT staff, is of utmost importance to ensure the applicability of a to-be-developed CDM. In addition, user-friendly, adaptable, and straightforward models are preferred in health care as one can start working with them without requiring a substantial amount of time [84].

This scoping review provides a summary of the development methods for CDMs and categorizes them based on the requirement analysis method, design process, validation approach, and model type. A variety of methods were used in the requirement analysis step in the articles, starting from searching in different types of literature and medical guidelines [43,44] to interviews [29], the Delphi approach [31], and a review of existing CDEs. A full list of these articles is shown in Figure 4 and Multimedia Appendix 4.

The majority of the developed CDMs have been designed for common medical conditions, and only 10 articles involved the design of a particular CDM for rare diseases. However, we did not find a significant difference in the development process of a CDM for rare and common conditions. Interestingly, based on our analysis, we can conclude that common medical conditions were the focus of CDM studies from early 2000, whereas the first CDM for rare conditions was developed in 2014. Despite methodological similarities, every article usually mentioned following a more individualistic method of development. This may arise because rare conditions occur rarely and the number of patients included in studies is limited [44]. Moreover, finding an expert for each rare or unclear disease is a challenging task. Additionally, most of the information crucial in the diagnosis of such diseases (like symptoms or phenotypes and genotypes) is currently stored in unstructured forms (eg, clinical notes). Extraction of such information requires a lot of time and effort from technical and clinical stakeholders [41].

Thus, given the variety of studies, the methods used for common conditions might be adaptable for rare conditions. Considering that a CDM is an essential part of data harmonization (a necessity in the health domain), we see highly emphasized development models as essential. Therefore, after analyzing the included CDMs, we summarized a suggestive development process that is shown in Figure 5, which could be the starting point for conceptualizing and implementing novel CDMs.

### Limitations

The findings of our study are subject to certain limitations. First, our analysis is restricted to the selected databases, namely, PubMed, Web of Science, Science Direct, and Scopus. Additionally, the scope of our investigation is confined to articles published within a specific time frame and written in English. Moreover, we did not conduct any assessment of the quality of the included articles. In addition, it may also be worth noting that the authors of this review have varying interdisciplinary backgrounds, expertise levels, and experiences in the CDM field. However, to optimize the screening and analyzing processes, we performed them in pairs and first tested the method on a subset of 10% of the articles, resulting in a minimal number of conflicts.

### Conclusion

We considered 4 steps in the development of a CDM: conception, users’ needs collection, implementation, and evaluation. We could identify 4 groups of methods that were most often used in the articles as part of the requirement analysis of the CDM development process. These were literature analysis, interviews, Delphi approaches, and review of existing CDEs. The articles considered in this review either developed a new CDE or made use of an existing set of CDEs with or without modification.

Most of the articles involved at least one stakeholder from among domain experts, clinicians, IT staff, registry staff, and patients’ representatives, and mostly from the initial step, which was conception. The methods used to involve the stakeholders were expert groups, surveys, consensus meetings, interviews, working groups, teleconferences, questionnaires, and workshops, and among these, working groups were most often used.

We conclude that the methods used in the development of CDMs in the health domain are heterogeneous and this field is lacking solid guidelines that may ease up this process, especially in terms of the reusability and adaptability of a CDM. This is why the proposed outline (Figure 5) could be a reasonable basis to start with. In our future work, we plan to test and improve the proposed outline for developing a CDM.

## Appendix

Multimedia Appendix 1 PRISMA-ScR (Preferred Reporting Items for Systematic Reviews and Meta-Analyses extension for Scoping Reviews) checklist.

Multimedia Appendix 2 Search strings used in the PubMed, Web of Science, Science Direct, and Scopus databases to search for articles.

Multimedia Appendix 3 Inclusion and exclusion criteria for the title and abstract screening, and the full-text screening.

Multimedia Appendix 4 Additional study information.

Multimedia Appendix 5 Characteristics of the included studies.

Multimedia Appendix 6 Summary of derived stakeholder information.

## Abbreviations

CDE: common data element
CDM: common data model
MeSH: Medical Subject Headings
OMOP: Observational Medical Outcomes Partnership
PRISMA-ScR: Preferred Reporting Items for Systematic Reviews and Meta-Analyses extension for Scoping Reviews
SATURN: Smartes Arztportal für Betroffene mit unklarer Erkrankung (Smart physicians’ platform for patients with unclear diseases)

## Data Availability

The script used for analysis and visualization in this review is available at GitHub [26], and the study protocol can be accessed on the Open Science Framework (OSF) [19].
