# Supplementary material for: Methods Used in the Development of Common Data Models for Health Data: Scoping Review
Source: JMIR Med Inform. 2023 Aug 3;11:e45116. doi: 10.2196/45116 (PMC10436118; doi:10.2196/45116)
Supplement: Multimedia Appendix 2 [file medinform_v11i1e45116_app2.docx]

| Database | Search string | Limitations |
| --- | --- | --- |
| PUBMED | ((("common data model"[tw] AND "CDM"[tw]) OR ("common data element*"[tw] AND CDE [tw]) OR "Common Data Elements"[Mesh] OR "common dataset*"[tw] OR "common data set" [tw]) AND (Medical OR Medicine OR "Medicine"[Mesh] OR health OR "Health"[Mesh] OR healthcare OR "health care" OR "electronic health" OR clinical OR disease* OR "Disease"[Mesh])) AND (("2000/01/01"[Date - Publication]: "2022/03/15"[Date - Publication])) | Date: 2000-2022 |
| Web of Science | (((((TI=(("common data model" AND CDM) OR ("common data element" AND CDE) OR ("common dataset*" OR "common data set*"))) OR AB=(("common data model" AND CDM) OR ("common data element" AND CDE) OR ("common dataset*" OR "common data set*"))) OR AK=(("common data model" AND CDM) OR ("common data element" AND CDE) OR ("common dataset*" OR "common data set*"))) OR KP=(("common data model" AND CDM) OR ("common data element" AND CDE) OR ("common dataset*" OR "common data set*"))) AND ALL=(medical  OR  medicine  OR  health*  OR  healthcare  OR  "health care"  OR  "electronic health"  OR  clinical  OR  disease)) AND DOP=(2000-01-01/2022-03-15) | Date: 2000-2022 |
| Science Direct | Articles with these terms: (Medical OR Medicine OR health OR healthcare OR “health care” OR “electronic health” OR clinical OR disease) AND Title, abstract or author specified keywords: (("common data model" AND CDM) OR ("common data element" AND CDE) OR ("common dataset" OR "common data set")) | Date: 2000-2022 |
| SCOPUS | ( TITLE-ABS-KEY ( ( "common data model"  AND  cdm )  OR  ( "common data element"  AND  cde )  OR  ( "common dataset"  OR  "common dataset" ) )  AND  ALL ( ( medical  OR  medicine  OR  health*  OR  healthcare  OR  "health care"  OR  "electronic health"  OR  clinical  OR  disease ) ) )  AND  ( LIMIT-TO ( PUBYEAR ,  2022 )  OR  LIMIT-TO ( PUBYEAR ,  2021 )  OR  LIMIT-TO ( PUBYEAR ,  2020 )  OR  LIMIT-TO ( PUBYEAR ,  2019 )  OR  LIMIT-TO ( PUBYEAR ,  2018 )  OR  LIMIT-TO ( PUBYEAR ,  2017 ) )  OR ( LIMIT-TO ( PUBYEAR ,  2016 )  OR  LIMIT-TO ( PUBYEAR ,  2015 )  OR  LIMIT-TO ( PUBYEAR ,  2014 )  OR  LIMIT-TO ( PUBYEAR ,  2013 )  OR  LIMIT-TO ( PUBYEAR ,  2012 )  OR  LIMIT-TO ( PUBYEAR ,  2011 )  OR  LIMIT-TO ( PUBYEAR ,  2010 )  OR  LIMIT-TO ( PUBYEAR ,  2009 )  OR  LIMIT-TO ( PUBYEAR ,  2008 )  OR  LIMIT-TO ( PUBYEAR ,  2007 )  OR  LIMIT-TO ( PUBYEAR ,  2006 )  OR  LIMIT-TO ( PUBYEAR ,  2005 )  OR  LIMIT-TO ( PUBYEAR ,  2004 )  OR  LIMIT-TO ( PUBYEAR ,  2003 )  OR  LIMIT-TO ( PUBYEAR ,  2002 )  OR  LIMIT-TO ( PUBYEAR ,  2001 )  OR  LIMIT-TO ( PUBYEAR ,  2000 ) )  AND  ( LIMIT-TO ( LANGUAGE ,  "English" ) ) | Date: 2000-2022 |

**Multimedia Appendix 2.** Search strings used in the PubMed, Web of Science, Science Direct, and Scopus databases to search for articles.
