# Supplementary material for: Methods Used in the Development of Common Data Models for Health Data: Scoping Review
Source: JMIR Med Inform. 2023 Aug 3;11:e45116. doi: 10.2196/45116 (PMC10436118; doi:10.2196/45116)
Supplement: Multimedia Appendix 3 [file medinform_v11i1e45116_app3.docx]

| Screening round | Inclusion | Exclusion |
| --- | --- | --- |
| Title and abstract screening | The article is primary research in a peer-reviewed journal or conference. | The article is of any other type. For instance, literature review, study protocols, commentaries and editorials, tutorials, project reports, medical case studies, and master and doctoral thesis. |
| Title and abstract screening | The article is written in English. | The article is written in a language other than English. |
| Title and abstract screening | The title or abstract focuses on a CDM in medical domain. | The title or abstract does not focus on a CDM in medical domain. |
| Full-text screening | The article defines CDM as a common data model for health data. | The article defines CDM differently than the common data model for health data. |
| Full-text screening | The article is defining (at least briefly) the development process, especially design or validation of a CDM in the medical domain. | The article only mentions a CDM in the medical domain without explaining the development process, design or validation of the CDM. |

**Multimedia Appendix 3.** Inclusion and exclusion criteria for the title and abstract screening, and the full-text screening.
