# Supplementary material for: Methods Used in the Development of Common Data Models for Health Data: Scoping Review
Source: JMIR Med Inform. 2023 Aug 3;11:e45116. doi: 10.2196/45116 (PMC10436118; doi:10.2196/45116)
Supplement: Multimedia Appendix 5 [file medinform_v11i1e45116_app5.pdf]

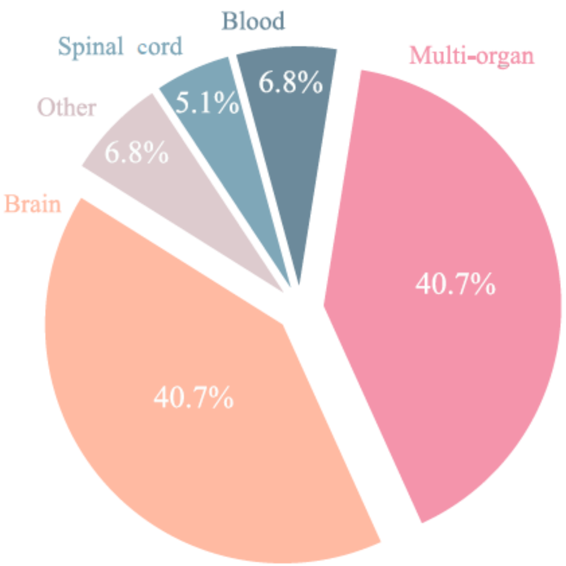


Figure S1

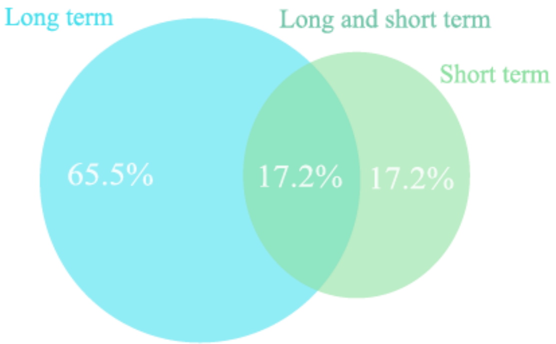


Figure S2

**Multimedia Appendix 5.** Characteristics of the included studies. Figure S1: A pie chart categorizing the medical conditions from the articles according to the organ that the condition usually affects. Figure S2: A Venn diagram showing the percentage of long-term versus short-term conditions and those that could be considered long term or short term.
