# Supplementary material for: Methods Used in the Development of Common Data Models for Health Data: Scoping Review
Source: JMIR Med Inform. 2023 Aug 3;11:e45116. doi: 10.2196/45116 (PMC10436118; doi:10.2196/45116)
Supplement: Multimedia Appendix 6 [file medinform_v11i1e45116_app6.pdf]

Figure S1

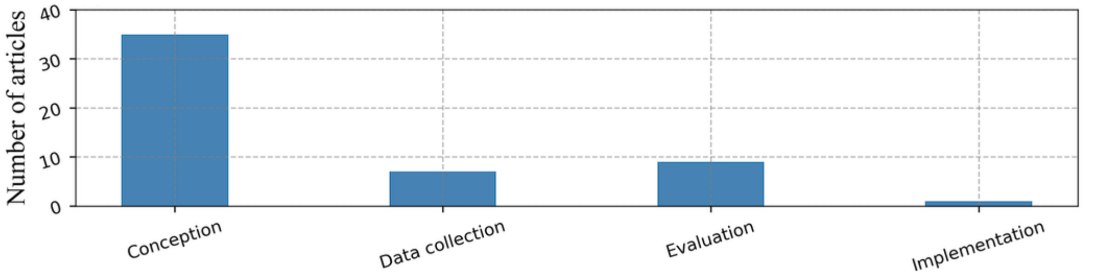

Figure S2

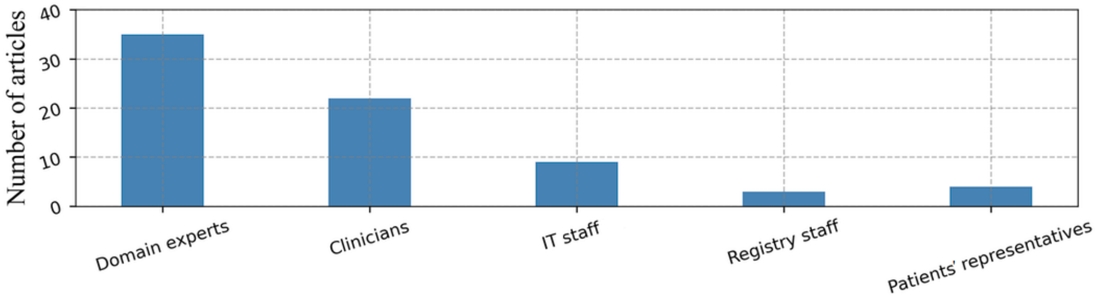

Figure S3

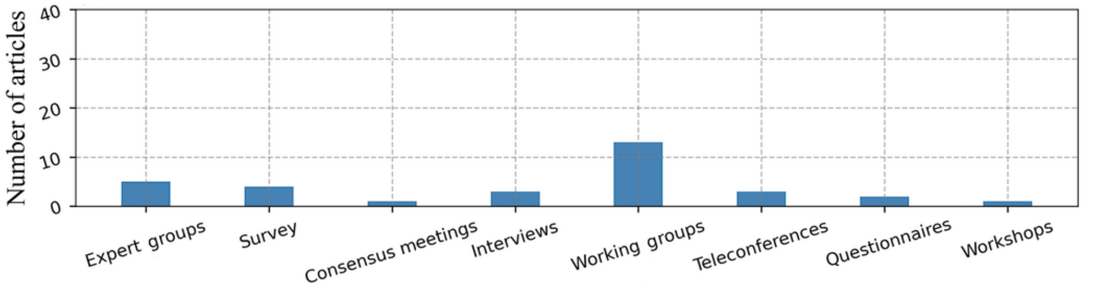

Multimedia Appendix 6. Summary of derived stakeholder information. Figure S1: Steps of the design process in which stakeholders were involved. In most articles, stakeholders were involved for the first time during the conception phase. Figure S2: Stakeholders who were involved in the design process itself. Figure S3: Nature of stakeholder involvement (eg, via a questionnaire or online survey).
